# The Association between the Dietary Inflammatory Index and Thyroid Function in U.S. Adult Males

**DOI:** 10.3390/nu13103330

**Published:** 2021-09-23

**Authors:** Nuozhou Liu, Fang Ma, Ying Feng, Xue Ma

**Affiliations:** 1 West China School of Medicine, West China Hospital, Sichuan University, Chengdu 610041, China; liunuozhou@stu.scu.edu.cn; 2 Center for Translational Medicine, Key Laboratory of Birth Defects and Related Diseases of Women and Children (Sichuan University), Ministry of Education, West China Second University Hospital, Sichuan University, Chengdu 610041, China; mafangmed@126.com; 3 Department of Obstetrics and Gynecology, West China Second University Hospital, Sichuan University, Chengdu 610041, China; 4 West China School of Basic Medical Sciences & Forensic Medicine, Sichuan University, Chengdu 610041, China; 5 Department of Pediatric Urology, West China Hospital, Sichuan University, Chengdu 610041, China

**Keywords:** diet, thyroid, inflammation, dietary inflammatory index, NHANES

## Abstract

Thyroid function has a close link with inflammation. However, it is still unknown whether the dietary inflammatory potential is associated with thyroid function. We aimed to assess the relationship among them using the data from the National Health and Nutrition Examination Survey (NHANES). This study was a cross-sectional study, where weighted multivariable linear regression, subgroup analyses, and interaction terms were employed. Thyroid function was assessed by eight indexes, including total and free T4 and T3, Tg, TgAb, TPOAb, and TSH. A total of 2346 male participants aged ≥20 years with an average age of 50.74 ± 17.68 years were enrolled. The mean DII score among participants was −0.46 ± 1.73, ranging from −4.12 to 4.41, and mean total thyroxine (T4) was 7.61 ± 1.51 μg/dL. We found a positive association between DII and total T4 (β = 0.07; *p* = 0.0044). Using subgroup analysis, this association became stronger in both the iodine-deficient and obese group (iodine-deficient group: β = 0.15, *p* < 0.0001; obese group: β = 0.14, *p* < 0.0001). In conclusion, men adhering to a more pro-inflammatory diet appeared to have higher total T4 levels. However, these hormone variations were still within the normal clinical range and more well-designed studies are still needed to validate the causal relationship between DII and thyroid function.

## 1. Introduction

The thyroid lies in the lower part of the front neck near the upper trachea. Under the regulation of the hypothalamic-pituitary-thyroid (HPT) axis, the organ secrets thyroid hormone, including triiodothyronine (T3) and tetraiodothyronine (thyroxine; T4) [1]. The majority of circulating T3 derives from T4 deiodination in peripheral tissue via iodothyronine deiodinases [2]. Both circulating T3 and T4 can tightly bind to plasma proteins, such as thyroxine-binding globulin (TBG) and transthyretin (TTR), where only 0.02% of T4 and 0.3% of T3 are unbound and biologically active, called free T4 and free T3, respectively [2]. The adenohypophysis-synthesized thyroid-stimulating hormone (TSH) stimulates thyroid hormone synthesis and secretion [1]. Thyroglobulin antibodies (TgAb) and thyroid peroxidase antibodies (TPOAb) are associated with hypothyroidism and are found in autoimmune thyroid disease like Hashimoto thyroiditis (chronic lymphocytic thyroiditis) [3].

Previous studies revealed that thyroid hormones (T3 and T4) were essential for growth, normal development, neural differentiation, and metabolic regulation in mammals [4,5,6]. Recently, the prevalence of hypothyroidism in different population surveys was reported to be just around 8–15%, increasing with age [7]. Abnormal thyroid hormone variation (not necessarily pathology) may result in poorer cognitive outcomes, secondary osteoporosis with increased fracture risk, and varying degrees of goiter [8,9].

The dietary inflammatory index (DII) is a literature-derived dietary tool for measuring individual dietary inflammatory potential proposed by J.R. Hébert et al. [10]. A more pro-inflammatory diet (higher DII score) predicts an increased level of interleukin-6 (IL-6) and C-reactive protein (CRP) [11,12]. Many disease occurrences and developments, including esophageal squamous cell cancer (ESCC) [13], squamous cell head and neck cancer [14], cardiovascular disease (CVD) [15], and testosterone deficiency [16], are positively associated with higher DII scores. Urine iodine concentration (UIC) is an assessment method of human iodine condition, which affects thyroid function significantly [17]. However, the relationship between DII and thyroid function is not clear.

As we mentioned before, a higher DII score indicated higher dietary inflammatory potential with increased pro-inflammatory markers. Previous studies revealed that inflammation might associate with thyroid function. The production of inflammatory cytokines from thyroid-infiltrating mononuclear and follicular cells was associated with Graves disease [18]. Systemic inflammation defined by monocyte-to-high-density-lipoprotein-cholesterol ratio (MHR) might be involved in the development of thyroid nodules (T.N.s) [19]. Leukocytic infiltration mainly constituted by macrophages (inflammation) was normal in transgenic mice expressing RET/PTC3 (RP3, an oncogenic fusion protein in the thyroid gland), with papillary thyroid carcinoma (PTC)-like lesions [20]. IL-21 and IL-21R were up-regulated in autoimmune thyroid disease (AITD), which is mainly known as Graves disease (G.D.) and Hashimoto thyroiditis (H.T.) [21]. The fibrotic process of the thyroid gland was observed in selenium-deficient thyroids with inflammatory reactions marked by macrophages [22]. A cross-sectional study confirmed that hemodialyzed (H.D.) patients with inflammatory complications showed thyroid hormone variation [23].

We aimed to explore the association between the dietary inflammatory condition and thyroid function via obtaining data from the National Health and Nutrition Examination Survey (NHANES). As we hypothesized, a pro-inflammatory diet might change the thyroid hormone profile or even harm the thyroid gland. This study might provide new insights on thyroid condition management from a clinical-nutritional perspective.

## 2. Materials and Methods

### 2.1. Study Population

The National Health and Nutrition Examination Survey (NHANES) is a nationwide and ongoing cross-sectional survey managed by the National Center for Health Statistics at the U.S. Centers for Disease Control and Prevention. It conducts a repeated 2-year cycle test with a complex multistage probability sampling design, and all sample weights are designed to represent data for the civilian noninstitutionalized U.S. population. This database includes five sections (Demographics, Dietary, Examination, Laboratory, and Questionnaire). The NHANES research protocol was approved by the institutional review board and included the written, informed consent of all participants, following the principles of the Declaration of Helsinki. Data in this study were all obtained from NHANES, publicly available at https://www.cdc.gov/nchs/nhanes/ (accessed date: 5 August 2021).

Our study was based on a 2-year NHANES survey cycle, 2007–2008, since this cycle included full information on UIC, thyroid function, and dietary condition to calculate DII. After excluding female participants (*n* = 2910), age < 20 years (*n* = 1210), participants without necessary dietary conditions (*n* = 135), thyroid function test results (including people with thyroid diseases), and UIC (*n* = 316), we finally selected 2346 male participants (age ≥ 20) in the 2007–2008 NHANES (Figure S1).

### 2.2. Exposure and Outcome Definitions

DII was designed as the exposure variable. Dietary intake was documented and validated utilizing the 24-h dietary history interview. The dietary data were obtained at the mobile examination center and were validated by the Nutrition Methodology Working Group [24]. The 24-h dietary recall data were used to calculate the DII score according to the calculating protocol published by N. Shivappa et al. [25] (see Supplementary Table S2). A higher positive DII score indicated a more pro-inflammatory diet, while a lower negative DII score suggested an anti-inflammatory effect of diet [25]. In our study, 28 of the 45 food parameters were available to calculate DII in the 2007–2008 NHANES cycle, including alcohol, protein, fiber, β-carotene, cholesterol, carbohydrates, energy, fats, n-3 fatty acid, n-6 fatty acid, poly-unsaturated fatty acid, mono-unsaturated fatty acid, saturated fat, thiamin, magnesium, zinc, selenium, iron, riboflavin, folic acid, vitamin A, vitamin B-6, vitamin B-12, vitamin C, vitamin D, vitamin E, caffeine, and niacin (see Supplementary Table S2). Previous studies revealed that using 27 or 28 food parameters would not affect the DII predictive capacity [26,27]. In this study, the DII score was served as a continuous variable and then categorized into continuous tertiles 1, 2, and 3 from the total sample for further analysis.

According to “Thyroid profile” in laboratory data from 2007–2008 NHANES, the measurement of thyroid function contained total and free Thyroxine (T4), total and Free Triiodothyronine (T3), thyroglobulin (Tg), thyroglobulin antibodies (TgAb), thyroid peroxidase antibodies (TPOAb), and thyroid-stimulating hormone (TSH) https://www.cdc.gov/nchs/nhanes/ (accessed date: 5 August 2021). The measurement of total T3 and T4 and free T3 and T4 were competitive binding immune-enzymatic assays. For total T3 and T4, a stripping agent was first used to dissociate T3 and T4 from the binding proteins. Then, specific antibodies were added to the sample followed with chemiluminescent substrate Lumi-Phos™ 530. Lastly, a luminometer was employed to measure the sample, where results were determined from a stored, multi-point calibration curve, while the stripping agent would not be added, as for the free T3 and T4 test. TSH was measured by a two-site, immune-enzymatic (“sandwich”) assay (third generation) and determined by a multi-point calibration curve (Lumi-Phos™ 530 was added). TPOAb and TgAb were measured by a sequential two-step immunoenzymatic “sandwich” assay, while Tg assay was a simultaneous one-step “sandwich” assay (Lumi-Phos™ 530 was added). Urine iodine concentration (UIC) was measured by ICP-DRC-MS (Inductively Coupled Plasma Dynamic Reaction Cell Mass Spectroscopy) to determine iodine conditions in participants [17,28].

### 2.3. Study Covariates

We incorporated age (year), body mass index (BMI, kg/m^2^), Race/Ethnicity, education level, smoking status, urine iodine concentration (UIC), annual family income, U.S. military force experience (Y/N), marital status, energy intake (kcal), and protein intake (g) as our covariates. A previous study about DII categorized BMI into <25 (normal), 25–29.9 (overweight), and ≥30 kg/m^2^ (obese) for participants aged more than 20 [16,29], and we adopted this categorization. We divided UIC into UIC <100 (iodine deficient), 100–299 (normal), ≥300 μg/L (excessive iodine intake) to consider the iodine condition of participants [30], which would inevitably affect thyroid function. The smoking status was classified into never, former smoker, and current smoker based on the “Smoking—Cigarette Use” section in the NHANES Questionnaire data. A detailed procedure of classifying smoking status from NHANES has been reported elsewhere [31]. The detailed classification of education level, race/ethnicity, marital status, and annual family income are all available in Supplementary Table S1.

### 2.4. Statistical Analysis

We followed CDC guidelines when performing statistical analysis. Where suitable, sampling weights were employed and accounted for the complex multistage survey (sampling) design [32]. Categorical variables were presented as percentages, while continuous variables were presented as mean ± standard deviation (S.D.) in this study. We employed the weighted student’s test (for continuous variables) and the weighted chi-square test (for categorical variables) to measure differences grouped by the continuous three DII tertiles. Weighted multivariable linear regression was applied to investigate the association between DII and thyroid function (eight indexes were included: total and free T4 and T3, Tg, TgAb, TPOAb, and TSH). To further investigate the covariate influence on this association, we employed Model 1 (unadjusted), Model 2 (age, race, UIC, and smoking status were adjusted), and Model 3 (fully adjusted model). Considering BMI [33] and iodine condition [34] on thyroid function, the subgroup analyses by BMI and UIC categories were conducted. Additionally, when performing BMI or UIC subgroup analysis, all covariates except itself were adjusted. An interaction term was added to test the heterogeneity of associations between the subgroup BMI or UIC. The *p* < 0.05 with effective confidence interval (CI) was considered statistically significant. All statistical analyses were performed by the software package R (http://www.R-project.org, The R Foundation, access on 28 April 2021) and EmpowerStats (www.empowerstats.com access on 28 April 2021).

## 3. Results

### 3.1. Baseline Characteristics of Participants

Sociodemographic and dietary characteristics and related covariates’ and dependent variables based on DII tertiles are presented in Table 1 and Supplementary Table S1. After applying the selection and exclusion criteria, a total of 2346 adult men with an average age of 50.7 ± 17.7 and DII of −0.5 ± 1.7 were included in this study. Mean DII scores ranged from −4.12 (most anti-inflammatory) to 4.41 (most pro-inflammatory), whereas the three DII tertile ranges were −4.21~1.40, −1.40~0.21, and 0.21~4.41, respectively. Among the three DII tertiles, for dietary condition and laboratory examination, differences in mean energy and protein intake, TSH, and total T4 were statistically significant (*p* < 0.05). For sociodemographic information, the differences with statistical significance (*p* < 0.05) were race/ethnicity, smoking status, education level, marital status, and annual family income (Supplementary Table S1). Mean ± SD energy and protein intakes were 2401.04 ± 1149.41 kcal and 92.81 ± 47.49 g, respectively. Participants in tertile 3 (more pro-inflammatory diet) showed relatively lower energy and protein intakes (1729.43 ± 716.02 kcal and 64.08 ± 31.26 g, respectively). Furthermore, men in tertile 3 presented a higher percentage of obesity and current smoking compared with other tertiles, and Non-Hispanic Black seemed to be more likely to have a diet with a higher DII score. Mean ± SD total T4 was 7.61 ± 1.51 μg/dL, where men had an average total T4 of 7.79 ± 1.58 μg/dL in tertile 3 (highest DII score) and 7.44 ± 1.47 μg/dL in tertile 1 (lowest DII score) with statistical significance (*p* < 0.05). The average of TSH was 1.97 ± 2.02 mIU/L, and men in tertile 2 (middle DII score) tended to have a higher TSH level (2.14 ± 2.90 mIU/L) compared with tertiles 1 and 3 (*p* = 0.009). No significant differences were found among the DII tertiles in thyroglobulin antibodies, free T3, free T4, thyroglobulin, total T3, and TPOAb (all *p* > 0.05). However, total T4 and TSH variations stayed within the recommended clinical range.

### 3.2. The Relationship between DII and Thyroid Function in Males Aged More Than 20 Years

Weighted multivariable linear regression was employed to illustrate the association between DII and thyroid function for males more than 20 years (Table 2). Our results revealed that higher DII was associated with a slightly higher total T4 level within the recommended clinical range (Model 1, β = 0.09, 95% CI: 0.06, 0.13, *p* < 0.0001; Model 2, β = 0.09, 95% CI: 0.06, 0.13, *p* < 0.0001; Model 3, β = 0.07, 95% CI: 0.02, 0.11, *p* = 0.0044). There were also higher free T3 and T4 levels (β = 0.01, 95% CI: 0.01, 0.02, *p* = 0.0005; β = 0.04, 95% CI: 0.00, 0.09, *p* = 0.0388) associated with a higher DII score in model 2. As for free T3, this positive association remained in the fully adjusted model (Model 3, β = 0.02, 95% CI: 0.01, 0.03, *p* = 0.0006).

After DII was grouped as tertiles, this positive association between DII and total T4 became more evident. The effect size was 0.15 for tertile 2 (Model 1, β = 0.15, 95% CI: 0.00, 0.30, *p* = 0.0476), and 0.35 for tertile 3 (Model 1, β = 0.35, 95% CI: 0.20, 0.50, *p* < 0.0001). Moreover, the TSH level was higher (mode 1, β = 0.30, 95% CI: 0.10, 0.50, *p* < 0.0033) in the highest tertile of the DII score (most pro-inflammatory) compared with the most anti-inflammatory diet (tertile 1).

When we adjusted for age, race, UIC, and smoking status (Model 2), as we mentioned above, a statistically significant positive association remained in total T4 and free T3 and T4. Our results also revealed a strong positive association among DII and total T3 for DII tertile 3 (β = 3.08, 95% CI: 1.02, 5.13, *p* = 0.0034) compared with the most anti-inflammatory diet. It meant that each unit of DII increase was associated with 3.08 ng/dL increase in total T3 compared with the reference group (tertile 1). Moreover, this association remained remarkable when we utilized a fully adjusted model (Model 3), whose effect size was 3.99 (β = 3.99, 95% CI: 1.49, 6.49, *p* = 0.0018) for DII tertile 3. Interestingly, after DII was grouped into the three continuous tertiles, a statistically significant positive correlation between DII and TSH only existed in DII tertile 2, relative to the most anti-inflammatory diet (tertile 1). In contrast, effect sizes were 0.30 in model 1, 0.31 in model 2, and 0.32 in model 3 (all *p* < 0.05 and effective confidence interval). TSH levels remained within the normal clinical range, and we did not find any statistically significant correlation among DII and thyroglobulin antibodies, thyroglobulin, and TPOAb (all *p* > 0.05).

We employed two new subgroup analyses to clearly evaluate the relationship between DII and related thyroid function, which classified these relationships based on different BMI and UIC groups. The interaction term test also found no significant difference between DII and thyroid function among different BMI or UIC groups (all *p* for interaction >0.05), revealing that the heterogeneities among different subgroups were not statistically significant. After adjusting for all covariates except BMI (Table 3), two similar positive correlations among DII and total T4 for participants in the overweight and obese groups were observed (Overweight, β = 0.07, 95% CI: 0.01, 0.12, *p* = 0.0228; Obese, β = 0.14, 95% CI: 0.08, 0.20, *p* < 0.0001).

Furthermore, total T4 in the obese group showed a relatively strong positive relationship with DII. The effect size was 0.26 for tertile 2 (β = 0.26, 95% CI: 0.01, 0.51, *p* = 0.0476) and 0.61 for tertile 3 (β = 0.61, 95% CI: 0.37, 0.85, *p* < 0.0001), relative to the most anti-inflammatory diet (tertile 1). Additionally, in the obese group, free T3 was firstly found to be positively associated with DII score, whose effect size was 0.36 (β = 0.36, 95% CI: 0.08, 0.65, *p* = 0.0125) for the most pro-inflammatory diet (tertile 3) compared with the most anti-inflammatory diet (tertile 1). Meanwhile, a strong positive relationship between DII and TPOAb was found in the obese but did not meet the statistical significance. Consistent with the results before BMI subgroup analysis, associations between DII and thyroglobulin antibodies and thyroglobulin were not statistically significant. Then, we adjusted for all covariates except UIC (Table 4) to attenuate iodine intake dissimilarity among different DII tertiles.

Surprisingly, for participants classified as iodine deficient, a positive association among total T4 and DII was more clear (β = 0.15, 95% CI: 0.08, 0.21, *p* < 0.0001). After DII was divided into tertiles, this positive association remained, whose effect size was 0.36 for tertile 2 (β = 0.36, 95% CI: 0.07, 0.65, *p* = 0.0148) and 0.65 for tertile 3 (β = 0.65, 95% CI: 0.37, 0.93, *p* < 0.0001) in relation to tertile 1 (the most anti-inflammatory diet). Free T4 level also showed a similar relationship with DII. Compared with DII tertile 1 (the most anti-inflammatory diet), the effect sizes (with statistical significance) were larger for tertile 2 (0.37) than that for tertile 3 (0.33). In the normal group, only total T4 showed a positive relationship with DII (β = 0.08, 95% CI: 0.03, 0.13, *p* = 0.0027). Additionally, total T4 was obviously higher among those in the highest tertile of the DII score (most pro-inflammatory diet) relative to those with the most anti-inflammatory diet (β of tertile 3 = 0.27, 95% CI: 0.06, 0.49, *p* = 0.0116). However, all outcome variables to evaluate thyroid function did not reach statistical significance in the excessive iodine intake group. Our findings revealed that a pro-inflammatory diet (higher DII score) might lead to higher TSH, total T3 and T4, free T3 and T4 levels. Additionally, after subgroup analysis, the original positive association among DII and total T3 and T4 and free T3 and T4 might be stronger in the overweight and obese groups and iodine-deficient group, while excessive iodine intake might attenuate these alternations.

## 4. Discussion

Our study aimed to assess the relationship between DII and thyroid function with a multivariable linear regression model. We incorporated 2346 male participants (age ≥20) in this cross-sectional study, where a positive association among a pro-inflammatory diet and TSH and total T4 was revealed as statistically significant. In the adjusted model, a higher total T3 level was observed in DII tertile 3 (pro-inflammatory diet) compared with an anti-inflammatory diet (DII tertile 1). Subgroup analysis stratified by BMI showed that a total T4 level might increase more dramatically in obese participants with higher DII scores. At the same time, free T3 and T4 levels also became positively associated with dietary inflammatory potential. We also conducted a UIC-stratified subgroup analysis. This subgroup analysis suggested a more significant positive correlation between dietary inflammatory potential and total T4, free T3 and T4, and TSH in the iodine-deficient group, while these differences became non-statistically significant in the excessive iodine intake group. Although we found a statistically significant difference in these hormones above, these variations were still within the normal clinical range, which probably meant that a pro-inflammatory diet had an uncertain association with thyroid abnormality. In both BMI and UIC subgroup analysis, interaction terms were employed and suggested that BMI or UIC did not influence the aforementioned associations.

To our knowledge, this is the first cross-sectional study investigating an association between dietary inflammatory potential and thyroid function. As we all know, iodine intake significantly affects thyroid function. Nevertheless, several epidemiological studies have found that diet intake (not about iodine) could also affect thyroid function. G. Liu et al. reported that overweight and obese participants with higher baseline free T3 and free T4 predicted more weight loss when on a weight-loss diet in a 2-year cycle [35]. Zn alone or combined with Se might also influence thyroid function, where mean serum-free T3 increased significantly [36]. Researchers enrolled 15 healthy male volunteers, suggesting that T3 and T3/rT3 ratios decreased after hypocaloric diet or metformin supplementation [37]. Jane Teas et al. found seaweed supplementation led to a higher TSH level, while soy supplementation did not influence thyroid function end points [38]. When comparing an isocaloric diet with high poly-unsaturated fat (H.F.) and high protein (H.P.), T3 declined more following the H.F. diet than the H.P. diet, while T4 and rT3 were not altered significantly [39]. A randomized, parallel, placebo-controlled human intervention study revealed that palmaria palmata, which stimulates inflammation, induced TSH variations within the normal clinical range [40]. However, a black pepper-based beverage (BPB) consisting of 20 mg gallic acid equivalent (GAE) attenuated hunger but did not alter T3 and T4 levels, as people had hypothesized [41].

The results of the BMI-stratified subgroup analysis indicated that the positive association between dietary inflammatory potential and thyroid function became more evident in overweight and obese males. Previous studies had done much work on thyroid function under the impact of being overweight or obesity. Roberta Zupo et al. conducted a cross-sectional study exploring the relationship between skeletal muscle mass (MM) and thyroid function in overweight and obese people and found that MM could enhance thyroid activity in obese people with increased T4 conversion to T3 and higher FT3 circulating levels, especially in men [42]. Further, their work reflected the possibility of a biological modulation to attenuate overfeeding-induced weight gain by stimulating thermogenesis via enhancing thyroid function [42]. In addition, a population-based prospective study indicated that higher free T3 level was a consequence rather than a cause of weight gain [43]. Another cross-sectional study based on a cohort of 778 euthyroid subjects found a positive relationship between serum TSH and BMI, which correlated well with our findings [44]. Cari M. Kitahara et al. utilized NHANES data and found that BMI and waist circumference were positively associated with serum TSH levels and free T3 but not free T4 among euthyroid adults [45]. A recent cohort study based on 1564 Chinese participants confirmed that overweight and obesity might lead to higher free T3 levels [46]. The underlying mechanism for higher T3 and serum TSH levels was not clear. A review noted that leptins, secreted by adipose cells, promoted thyrotropin-releasing hormone (TRH) gene expression directly in the paraventricular nucleus and ultimately stimulated TSH synthesis and secretion [33]. Leptin might also contribute to T4 to T3 conversion by deiodinases [33]. Thyroid function might also have an impact on adipose tissue distributions. A cross-sectional study that recruited 303 healthy individuals demonstrated that increased abdominal subcutaneous fat accumulation was associated with lower free T4 and higher TSH levels among euthyroid, slightly overweight individuals [47]. Additionally, this distribution difference was probably mediated by a differential TSH receptor and/or thyroid hormone receptor expression in different fat depots [33].

Our results’ underlying mechanism behind the positive relationship between DII score (dietary inflammatory potential) and thyroid function variation was unclear. Based on the DII calculation protocol, we categorized food components into pro-inflammatory and anti-inflammatory to address how dietary components affect thyroid function. As for cholesterol, a pro-inflammatory food parameter, a meta-analysis found that treating overt hyperthyroidism led to a significant decline in total cholesterol [48]. Animal studies confirmed that soy protein supplementation, which could decrease serum cholesterol levels, would lead to higher T4 levels [49]. Vitamin B12, with an overall inflammatory effect score >0, is defined as a pro-inflammatory food parameter in the dietary inflammatory index calculation. Patients with autoimmune thyroid disease (AITD) are more susceptible to Vitamin B12 deficiency and pernicious anemia [50]. Thyroid hormones also play an essential role in synthesizing flavine mono- and di-nucleotides from vitamin B12 [51]. The prevalence of vitamin B12 deficiency or insufficient intake in thyroid-disorder patients is relatively frequent [52,53], and vitamin B12 might promote thyroid hormone synthesis. A review conducted by Josef Kohrle concluded that selenium (an anti-inflammatory food component) supply was essential to ensure a normal thyroid hormone profile and preserve the integrity of the thyroid gland exposed to H_2_O_2_, excess stimulation by thyrotropin (TSH) or TSH receptor-stimulating antibodies (TRAK), or attack by the immune system in autoimmune thyroiditis [54]. Additionally, impaired selenium status might cause dysfunction of thyroid hormone synthesis and metabolism [54]. Zinc, an anti-inflammatory microelement, was also associated with thyroid hormone profiles. Zn-deficient animals showed depletion of serum T3 and T4 with changes of atrophy and degeneration of thyroid follicles [55,56]. However, a recent systematic review pointed out that Zn supplementation’s impact on thyroid function might depend on patients suffering from some kind of disease, which could not be simply generalized to the average human population [57]. For instance, a negative correlation between Zn concentration and total T3 and T4 level was found in hyperthyroid and primary hypothyroid patients, while this association became controversial in individuals without thyroid disease [57]. Vitamin C (anti-inflammatory) supplementation could attenuate the degeneration of the albino rat thyroid gland induced by oral monosodium glutamate and kill thyroid cancer cells by inhibiting MAPK/ERK and PI3K/AKT pathways via a ROS-dependent mechanism [58,59]. However, a clinical trial identified that Vitamin C and E supplementation might not change free T3 and T4 levels in postmenopausal women with type 2 diabetes [60].

Some evidence also showed that probable mechanisms for T3 and T4 variation could be the effect of diet on pro-inflammatory markers such as IL-1, IL-6, IL-17, and TNF. For instance, surgery-induced rapid inflammatory response, characterized by activation of neutrophils and the release of various pro-inflammatory cytokines, could alter or even impair the serum thyroid hormone profile [61].

However, as for inflammatory cytokines, the mechanism became controversial or even contrary to our results. N.K. cells and macrophages were thought to play a central role in the secretion of IL-1 and IL-6, which can be potentiated by TNF-α. However, these markers’ up-regulation may suppress thyroid function instead of stimulating it, as in our results [62,63]. Furthermore, pro-inflammatory cytokines (IL-1a, IL-1b, IL-6, and TNF-a) may inhibit sodium–iodine symporter (NIS) protein expression and NIS gene transcription and then lead to various degrees of thyroid dysfunction [64,65]. These cytokines may also inhibit the synthesis and activity of Type Ⅰ 5′-deiodinase by competing with the coactivator of liver type I 5′-deiodinase gene expression, activating the transcription regulator NF-κB, etc., and then lead to a decrease in blood thyroid hormone levels [66]. However, these studies concentrated mainly on infection-related inflammation, especially in animal models, where pro-inflammatory cytokines increased too dramatically compared with diet-induced inflammatory cytokine ascent in humans. More diet-based studies on humans should explore a more convincing mechanism for dietary thyroid function variations.

Although variations in our results about thyroid-related indexes stayed within the normal clinical range, we still need to carefully consider our diet inflammatory potential to protect our thyroid gland. A similar cross-sectional study conducted in New Caledonia found strong positive associations between DII and thyroid cancer risk. The odds ratio reached 1.67 with statistical significance, representing that people in the extreme DII tertile had a significantly (67%) higher carcinoma risk than those in the lowest tertile [67]. Other studies also confirmed significant positive associations between levels of IL-6 and CRP and thyroid cancer based on a larger population [68,69]. However, IL-4 and IL-10, defined as anti-inflammatory cytokines in DII calculation, could pose both anti-cancer and pro-cancer actions in the tumor microenvironment depending on the specific molecular and cellular context [70]. We thought that dietary inflammatory potential might become more critical to people with the thyroid-related disease, which needed further investigation focusing on thyroid disease patients.

However, our study had some limitations. (1) The cross-sectional study design severely hindered our ability to make a causal inference. A subsequent, large-cohort study is important to confirm our results further. (2) DII calculations based on 24-h dietary recall, annual family income, and martial and smoking statuses were obtained from questionnaires in NHANES; hence, recall bias and social desirability bias are inevitable. (3) Due to thyroid gland development uncertainty and low questionnaire compliance in children, we excluded participants <20 yrs. More child-oriented studies should be done. (4) Due to the uncertain influence on thyroid function of woman in pregnancy and menstruation, we excluded female participants. (5) NHANES did not contain information on medications that could probably affect thyroid functions. (6) The insufficiency of candidate basic and clinical studies made it very hard to clearly explain the association between DII and related hormone fluctuations.

Nevertheless, this study is the first nationwide, cross-sectional study to evaluate the association between dietary inflammatory potential and thyroid function based on NHANES data.

## 5. Conclusions

Men with a more pro-inflammatory diet (higher DII score) showed higher total T4 levels, and this association might become stronger in men with obesity or iodine deficiency. Total T3 and TSH levels only became higher among participants with a more pro-inflammatory diet (tertile 3 or tertile 2) relative to those with the most anti-inflammatory diet (tertile 1). However, these hormone level variations were still within the normal clinical range and, thus, may have had an uncertain impact on disease risk. Our study indicated that dietary management might be necessary to ensure normal thyroid function in our lives. However, more well-designed studies are still needed to validate and verify the causal relationship between DII and thyroid function.

## Figures and Tables

**Table 1:** Baseline characteristics of participants in the 2007–2008 continuous NHANES.

	Total	DII Tertile 1	DII Tertile 2	DII Tertile 3	*p*-Value
Participant number	2346	782	782	782	
Mean ± SD age (yrs)	50.74 ± 17.68	49.77 ± 17.33	51.58 ± 17.50	50.87 ± 18.19	0.124
Mean ± SD DII	−0.46 ± 1.73	−2.31 ± 0.63	−0.61 ± 0.46	1.54 ± 0.95	<0.001
Mean ± SD Energy (kcal)	2401.04 ± 1149.41	3111.88 ± 1350.96	2361.82 ± 818.46	1729.43 ± 716.02	<0.001
Mean ± SD Protein intake (g)	92.81 ± 47.49	91.74 ± 35.14	91.74 ± 35.14	64.08 ± 31.26	<0.001
Mean ± SD Thyroglobulin antibodies (IU/mL)	8.90 ± 90.91	8.02 ± 94.45	11.28 ± 99.37	7.39 ± 77.54	0.663
Mean ± SD Free T3 (pg/mL)	3.25 ± 0.39	3.24 ± 0.39	3.24 ± 0.39	3.26 ± 0.39	0.350
Mean ± SD Free T4 (pmol/L)	10.07 ± 1.79	10.05 ± 1.87	10.00 ± 1.74	10.14 ± 1.75	0.309
Mean ± SD Thyroglobulin (ug/L)	14.93 ± 52.98	12.96 ± 17.55	15.09 ± 49.41	16.74 ± 75.31	0.368
Mean ± SD TSH (mIU/L)	1.97 ± 2.02	1.84 ± 1.19	2.14 ± 2.90	1.92 ± 1.53	0.009
Mean ± SD Total T4 (μg/dL)	7.61 ± 1.51	7.44 ±1.47	7.59 ± 1.46	7.79 ± 1.58	<0.001
Mean ± SD Total T3 (ng/dL)	113.07 ± 21.88	111.92 ± 21.63	112.96 ± 21.98	114.34 ± 22.00	0.090
Mean ± SD Thyroid peroxidase antibodies (IU/mL)	11.54 ± 67.98	9.13 ± 52.73	13.52 ± 74.72	11.99 ± 74.17	0.432
Race/Ethnicity (%) Mexican American Other Hispanic Non-Hispanic White Non-Hispanic Black Other		17.8 9.6 53.8 13.9 4.9	18.0 9.2 51.2 16.5 5.1	15.0 11.3 44.8 26.2 2.8	<0.001
Smoking status (%) Never Former smoker Current smoker Missing		47.8 28.8 23.1 0.3	40.4 34.5 25.1 0	39.6 32.9 27.5 0	0.005
BMI (%) Normal (<25 kg/m^2^) Overweight (25–29.9 kg/m^2^) Obese (≥30 kg/m^2^)		30.2 37.5 32.4	26.3 41.0 32.6	25.2 37.6 37.2	0.061
UIC * (%) Iodine deficient (<100 ug/L) Normal (100–299 ug/L) Excessive iodine intake (≥300 ug/L)		25.8 50.4 23.8	23.3 53.6 23.1	26.3 50.1 23.5	0.579

For continuous variables, *p* value was calculated by weighted *t*-test. For categorical variables, *p*-value was calculated by weighted chi-square test. * UIC stands for urine iodine concentration to evaluate iodine intakes.

**Table 2:** Association between Dietary Inflammatory Index and thyroid function among U.S. adult men in NHANES from 2007 to 2008.

DII Tertile	TgAb (IU/mL)	Free T3 (pg/mL)	Free T4 (pmol/L)	Tg (ug/L)	TSH (mIU/L)	Total T4 (μg/dL)	Total T3 (ng/dL)	TPOAb (IU/mL)
**β (95% CI), *p* Value**								
Model 1								
Continuous	−0.48 (−2.61, 1.65) 0.6589	0.01 (−0.00, 0.02) 0.0862	0.03 (−0.01, 0.07) 0.1946	1.20 (−0.04, 2.44) 0.0588	0.03 (−0.02, 0.08) 0.2476	0.09 (0.06, 0.13) <0.0001	0.71 (0.19, 1.22) 0.0070	0.31 (−1.29, 1.90) 0.7040
Tertile 1	Ref	Ref	Ref	Ref	Ref	Ref	Ref	Ref
Tertile 2	3.26 (−5.75, 12.27) 0.4784	0.00 (−0.04, 0.04) 0.9638	−0.05 (−0.22, 0.13) 0.5953	2.13 (−3.12, 7.38) 0.4274	0.30 (0.10, 0.50) 0.0033	0.15 (0.00, 0.30) 0.0476	1.03 (−1.13, 3.20) 0.3497	4.39 (−2.35, 11.13) 0.2018
Tertile 3	−0.62 (−9.64, 8.39) 0.8919	0.03 (−0.01, 0.06) 0.2015	0.09 (−0.09, 0.27) 0.3277	3.78 (−1.47, 9.03) 0.1585	0.08 (−0.12, 0.28) 0.4410	0.35 (0.20, 0.50) <0.0001	2.42 (0.25, 4.59) 0.0288	2.85 (−3.89, 9.59) 0.4070
Model 2								
Continuous	−0.22 (−2.38, 1.95) 0.8453	0.01 (0.01, 0.02) 0.0005	0.04 (0.00, 0.09) 0.0388	0.97 (−0.29, 2.23) 0.1310	0.04 (−0.00, 0.09) 0.0660	0.09 (0.06, 0.13) <0.0001	0.89 (0.40, 1.38) 0.0003	0.43 (−1.19, 2.05) 0.6013
Tertile 1	Ref	Ref	Ref	Ref	Ref	Ref	Ref	Ref
Tertile 2	3.74 (−5.30, 12.78) 0.4170	0.02 (−0.01, 0.05) 0.2250	−0.05 (−0.22, 0.13) 0.5970	1.42 (−3.85, 6.68) 0.5982	0.31 (0.11, 0.51) 0.0020	0.14 (−0.01, 0.29) 0.0694	1.67 (−0.36, 3.71) 0.1078	4.33 (−2.43, 11.10) 0.2097
Tertile 3	0.56 (−8.56, 9.68) 0.9039	0.05 (0.01, 0.08) 0.0055	0.16 (−0.02, 0.34) 0.0775	2.80 (−2.51, 8.11) 0.3018	0.1 (−0.1, 0.3) 0.1602	0.36 (0.21, 0.51) <0.0001	3.08 (1.02, 5.13) 0.0034	3.33 (−3.50, 10.15) 0.3398
Model 3								
Continuous	0.06 (−2.78, 2.90) 0.9651	0.02 (0.01, 0.03) 0.0006	0.02 (−0.04, 0.07) 0.5911	1.56 (−0.08, 3.21) 0.0631	0.14 (−0.06, 0.34) 0.1602	0.07 (0.02, 0.11) 0.0044	1.33 (0.70, 1.96) <0.0001	0.62 (−1.50, 2.75) 0.5650
Tertile 1	Ref	Ref	Ref	Ref	Ref	Ref	Ref	Ref
Tertile 2	4.50 (−5.27, 14.27) 0.3664	0.02 (−0.01, 0.06) 0.2186	−0.11 (−0.30, 0.08) 0.2519	2.20 (−3.46, 7.87) 0.4462	0.32 (0.11, 0.54) 0.0028	0.0 (−0.07, 0.25) 0.2967	2.01 (−0.16, 4.18) 0.0701	4.55 (−2.75, 11.85) 0.2220
Tertile 3	2.32 (−8.93, 13.56) 0.6866	0.05 (0.01, 0.09) 0.0117	0.03 (−0.18, 0.25) 0.7557	3.90 (−2.63, 10.42) 0.2420	0.09 (−0.16, 0.33) 0.4743	0.24 (0.06, 0.43) 0.0105	3.99 (1.49, 6.49) 0.0018	4.50 (−3.91, 12.90) 0.2946

Model 1: unadjusted; Model 2: age, race, UIC, and smoking status were adjusted; Model 3: age, race, smoking status, energy and protein intakes, education level, U.S. military force experience (Y/N), annual family income, UIC, BMI, and marital status were adjusted.

**Table 3:** Subgroup analysis of association between DII and thyroid function stratified by BMI groups among adult men in NHANES from 2007 to 2008.

DII Tertile	TgAb (IU/mL)	Free T3 (pg/mL)	Free T4 (pmol/L)	Tg (ug/L)	TSH (mIU/L)	Total T4 (μg/dL)	Total T3 (ng/dL)	TPOAb (IU/mL)
**β (95% CI2), *p* Value**								
Normal weight								
Continuous	−0.24 (−2.95, 2.46) 0.8610	−0.01 (−0.03, 0.01) 0.2728	−0.03 (−0.11, 0.06) 0.5428	0.57 (−1.73, 2.86) 0.6285	0.05 (−0.05, 0.16) 0.3145	0.06 (−0.01, 0.13) 0.1170	0.20 (−0.81, 1.21) 0.7039	−0.16 (−3.13, 2.81) 0.9160
Tertile 1	Ref	Ref	Ref	Ref	Ref	Ref	Ref	Ref
Tertile 2	−3.89 (−15.18, 7.40) 0.4997	−0.05 (−0.12, 0.03) 0.2164	−0.24 (−0.60, 0.12) 0.1979	4.74 (−4.81, 14.30) 0.3309	0.46 (0.02, 0.91) 0.0394	−0.07 (−0.38, 0.23) 0.6297	−0.79 (−5.01, 3.42) 0.7122	7.22 (−5.16, 19.60) 0.2535
Tertile 3	−1.22 (−12.65, 10.21) 0.8347	−0.04 (−0.12, 0.04) 0.2853	−0.20 (−0.57, 0.16) 0.2782	0.64 (−9.04, 10.31) 0.8970	0.13 (−0.32, 0.58) 0.5721	0.22 (−0.09, 0.53) 0.1664	0.63 (−3.64, 4.89) 0.7728	−0.69 (−13.22, 11.84) 0.9135
Overweight								
Continuous	−2.79 (−6.40, 0.81) 0.1293	0.02 (0.00, 0.03) 0.0204	0.04 (−0.03, 0.11) 0.2512	0.49 (−0.00, 0.98) 0.0514	0.0 (−0.02, 0.10) 0.6721	0.07 (0.01, 0.12) 0.0228	0.97 (0.14, 1.81) 0.0229	−0.77 (−3.42, 1.87) 0.5670
Tertile 1	Ref	Ref	Ref	Ref	Ref	Ref	Ref	Ref
Tertile 2	−2.78 (−17.61, 12.05) 0.7133	0.03 (−0.03, 0.09) 0.2974	−0.03 (−0.31, 0.25) 0.8450	0.87 (−1.14, 2.88) 0.3977	0.25 (−0.09, 0.60) 0.1530	0.22 (−0.01, 0.45) 0.0618	4.34 (0.91, 7.77) 0.0132	1.28 (−9.60, 12.15) 0.8182
Tertile 3	−10.18 (−25.33, 4.98) 0.1884	0.06 (0.00, 0.13) 0.0411	0.08 (−0.21, 0.36) 0.5974	0.05 (−0.30, 0.41) 0.7728	0.1 (−0.3, 0.4) 0.7728	0.20 (−0.04, 0.44) 0.0996	4.06 (0.56, 7.56) 0.0233	−1.27 (−12.38, 9.84) 0.8231
Obese								
Continuous	1.58 (−2.63, 5.78) 0.4627	0.01 (−0.00, 0.03) 0.1303	0.06 (−0.00, 0.13) 0.0613	2.32 (−0.72, 5.36) 0.1347	0.01 (−0.04, 0.07) 0.7004	0.14 (0.08, 0.20) <0.0001	0.79 (−0.06, 1.64) 0.0672	1.73 (−0.98, 4.43) 0.2110
Tertile 1	Ref	Ref	Ref	Ref	Ref	Ref	Ref	Ref
Tertile 2	15.94 (−2.61, 34.50) 0.0925	0.01 (−0.06, 0.07) 0.8611	0.11 (−0.18, 0.40) 0.4596	1.86 (−11.56, 15.27) 0.7863	0.20 (−0.04, 0.44) 0.1044	0.26 (0.01, 0.51) 0.0445	−1.35 (−5.11, 2.40) 0.4799	5.69 (−6.26, 17.63) 0.3511
Tertile 3	10.04 (−7.94, 28.01) 0.2740	0.03 (−0.03, 0.10) 0.3018	0.36 (0.08, 0.65) 0.0125	7.92 (−5.08, 20.91) 0.2329	0.03 (−0.21, 0.26) 0.8254	0.61 (0.37, 0.85) <0.0001	1.88 (−1.76, 5.51) 0.3116	9.50 (−2.08, 21.07) 0.1081
*p* for interaction	0.2279	0.0516	0.2334	0.3957	0.7557	0.1179	0.1249	0.4014

Subgroup analysis results were adjusted for age, race, smoking status, energy and protein intakes, education level, U.S. military force experience (Y/N), annual family income, UIC, and marital status. (BMI was not adjusted in this mode.)

**Table 4:** Subgroup analysis of the association between DII and thyroid function stratified by UIC groups among adult men in NHANES from 2007 to 2008.

DII Tertile	TgAb (IU/mL)	Free T3 (pg/mL)	Free T4 (pmol/L)	Tg (ug/L)	TSH (mIU/L)	Total T4 (μg/dL)	Total T3 (ng/dL)	TPOAb (IU/mL)
**β (95% CI2), *p* Value**								
Iodine deficient								
Continuous	−0.92 (−6.79, 4.94) 0.7575	0.01 (−0.01, 0.03) 0.3209	0.07 (−0.00, 0.14) 0.0614	0.57 (−0.09, 1.24) 0.0908	0.02 (−0.03, 0.08) 0.4323	0.15 (0.08, 0.21) <0.0001	0.98 (0.01, 1.95) 0.0486	−1.06 (−3.78, 1.67) 0.4474
Tertile 1	Ref	Ref	Ref	Ref	Ref	Ref	Ref	Ref
Tertile 2	3.41 (−22.76, 29.59) 0.7984	−0.02 (−0.10, 0.05) 0.5425	0.37 (0.04, 0.70) 0.0288	0.67 (−2.30, 3.63) 0.6597	0.27 (0.02, 0.51) 0.0324	0.36 (0.07, 0.65) 0.0148	−0.97 (−5.31, 3.38) 0.6632	11.20 (−0.91, 23.31) 0.0704
Tertile 3	−2.37 (−27.73, 22.99) 0.8550	0.02 (−0.05, 0.10) 0.5217	0.33 (0.01, 0.65) 0.0415	2.17 (−0.70, 5.04) 0.1395	0.09 (−0.15, 0.33) 0.4578	0.65 (0.37, 0.93) <0.0001	2.52 (−1.69, 6.73) 0.2409	−2.9 (−14.6, 8.8) 0.6271
Normal								
Continuous	−1.12 (−3.41, 1.17) 0.3372	0.01 (−0.00, 0.03) 0.0515	−0.00 (−0.06, 0.06) 0.9100	2.16 (−0.24, 4.55) 0.0778	0.01 (−0.04, 0.05) 0.7747	0.08 (0.03, 0.13) 0.0027	0.74 (0.02, 1.45) 0.0431	1.33 (−1.07, 3.73) 0.2775
Tertile 1	Ref	Ref	Ref	Ref	Ref	Ref	Ref	Ref
Tertile 2	3.48 (−5.99, 12.96) 0.4713	0.02 (−0.04, 0.07) 0.5109	−0.18 (−0.43, 0.07) 0.1486	3.90 (−6.02, 13.83) 0.4410	0.14 (−0.06, 0.34) 0.1613	0.12 (−0.09, 0.33) 0.2558	1.56 (−1.40, 4.52) 0.3030	−0.64 (−10.59, 9.30) 0.8994
Tertile 3	−3.36 (−12.99, 6.27) 0.4941	0.04 (−0.02, 0.09) 0.2089	−0.06 (−0.31, 0.20) 0.6577	6.61 (−3.48, 16.70) 0.1995	0.01 (−0.20, 0.21) 0.9610	0.27 (0.06, 0.49) 0.0116	5.91 (−4.20, 16.02) 0.2521	5.9 (−4.2, 16.0) 0.2521
Excessive Iodine Intakes								
Continuous	1.33 (−2.66, 5.31) 0.5144	−0.00 (−0.02, 0.02) 0.6609	0.04 (−0.05, 0.13) 0.3471	−0.11 (−1.00, 0.77) 0.7995	0.08 (−0.08, 0.25) 0.3183	0.06 (−0.02, 0.13) 0.1272	0.25 (−0.86, 1.37) 0.6534	−0.25 (−3.48, 2.97) 0.8774
Tertile 1	Ref	Ref	Ref	Ref	Ref	Ref	Ref	Ref
Tertile 2	3.35 (−13.27, 19.96) 0.6932	−0.01 (−0.09, 0.07) 0.7557	−0.19 (−0.56, 0.19) 0.3347	−0.48 (−4.17, 3.22) 0.7998	0.70 (0.02, 1.38) 0.0447	−0.00 (−0.32, 0.31) 0.9803	2.04 (−2.60, 6.67) 0.3899	8.93 (−4.52, 22.38) 0.1939
Tertile 3	7.05 (−9.50, 23.60) 0.4042	0.00 (−0.08, 0.08) 0.9207	0.13 (−0.25, 0.51) 0.4943	−0.45 (−4.13, 3.23) 0.8110	0.23 (−0.45, 0.90) 0.5144	0.17 (−0.14, 0.49) 0.2843	1.63 (−2.99, 6.24) 0.4907	2.81 (−10.59, 16.20) 0.6812
*p* for interaction	0.6542	0.3357	0.3183	0.3024	0.4482	0.2063	0.2697	0.4346

Subgroup analysis results were adjusted for age, race, smoking status, energy and protein intakes, education level, U.S. military force experience (Y/N), annual family income, BMI, and marital status. (UIC was not adjusted in this mode.)

## Data Availability

All data are available at NHANES websites https://www.cdc.gov/nchs/nhanes/ (accessed date: 5 August 2021).

## Supplementary Materials

The following are available online at https://www.mdpi.com/article/10.3390/nu13103330/s1. Figure S1: Flow diagram of the sample selection from NHANES 2007–2008. Table S1: Baseline characteristics of participants based on DII tertiles, weighted. Table S2: The methodology of dietary inflammatory index calculation.
