# Supplementary material for: The Association between the Dietary Inflammatory Index and Thyroid Function in U.S. Adult Males
Source: Nutrients. 2021 Sep 23;13(10):3330. doi: 10.3390/nu13103330 (PMC8540204; doi:10.3390/nu13103330)
Supplement: Supplementary file 1 [file nutrients-13-03330-s001.zip › Table S1ú║Baseline characteristics of participants based on DII tertiles, weighted..pdf]

Supplementary Table S1

Baseline characteristics of participants based on DII tertiles, weighted.

	Total	DII tertile 1 (-4.21~1.40)	DII tertile 2 (-1.40~0.21)	DII tertile 3 (0.21~4.41)	*P*-Value
Participant number	2346	782	782	782	
Mean±SD age (yrs)	50.74±17.68	49.77±17.33	51.58±17.50	50.87±18.19	0.124
Mean±SD DII	-0.46±1.73	-2.31±0.63	-0.61±0.46	1.54±0.95	<0.001
Mean±SD Energy (kcal)	2401.04±1149.41	3111.88±1350.96	2361.82±818.46	1729.43±716.02	<0.001
Mean±SD Protein intake (g)	92.81±47.49	91.74 ± 35.14	91.74±35.14	64.08 ± 31.26	<0.001
Mean±SD Thyroglobulin antibodies (IU/mL)	8.90±90.91	8.02±94.45	11.28±99.37	7.39±77.54	0.663
Mean±SD Free T3 (pg/ml)	3.25±0.39	3.24±0.39	3.24±0.39	3.26±0.39	0.350
Mean±SD Free T4 (pmol/L)	10.07±1.79	10.05±1.87	10.00±1.74	10.14±1.75	0.309
Mean±SD Thyroglobulin (ug/L)	14.93±52.98	12.96±17.55	15.09 ± 49.41	16.74 ± 75.31	0.368
Mean±SD TSH (mIU/L)	1.97±2.02	1.84±1.19	2.14±2.90	1.92±1.53	0.009
Mean±SD Total T4 (μg/dL)	7.61±1.51	7.44 ±1.47	7.59±1.46	7.79±1.58	<0.001
Mean±SD Total T3 (ng/dL)	113.07±21.88	111.92±21.63	112.96±21.98	114.34±22.00	0.090
Mean±SD Thyroid peroxidase antibodies (IU/ml)	11.54±67.98	9.13±52.73	13.52±74.72	11.99±74.17	0.432
Race/Ethnicity (%)					
Mexican American		17.8	18.0	15.0	
Other Hispanic		9.6	9.2	11.3	
Non-Hispanic White		53.8	51.2	44.8	<0.001
Non-Hispanic Black		13.9	16.5	26.2	
Other		4.9	5.1	2.8	
Smoking status (%)					
Never		47.8	40.4	39.6	0.005
Former smoker		28.8	34.5	32.9	

Current smoker	23.1	25.1	27.5	
Missing	0.3	0	0	
BMI (%)				
Normal (<25kg/m^2)	30.2	26.3	25.2	0.061
Overweight(25-29.9kg/m^2)	37.5	41.0	37.6	
Obese (≥30kg/m^2)	32.4	32.6	37.2	
UIC* (%)				
Iodine deficient (<100ug/L)	25.8	23.3	26.3	0.579
Normal (100-299ug/L)	50.4	53.6	50.1	
Excessive iodine intake (≥300 ug/L)	23.8	23.1	23.5	
Annual Family Income(%)				
$ 0--$ 4,999	1.7	1.4	3.1	<0.001
$ 5,000--$ 9,999	3.5	3.9	5.8	
$10,000--$14,999	4.8	8.0	8.7	
$15,000--$19,999	7.1	6.2	9.5	
$20,000--$24,999	7.6	9.4	11.2	
$25,000--$34,999	11.3	12.3	13.2	
$35,000--$44,999	8.4	10.1	8.9	
$45,000--$54,999	7.5	8.5	7.1	
$55,000--$64,999	5.8	6.1	6.0	
$65,000--$74,999	5.3	6.1	4.5	
$75,000--$99,999	9.9	8.8	6.2	
$100,000 and over	19.9	12.5	8.2	
Missing & unknown	2.3	3.0	3.3	
Education level (%)				
Less than 9th grade	10.1	12.3	20.5	<0.001
9-11th grade	14.2	16.8	19.6	
High school grade	20.2	28.1	26.6	
Some college or AA degree	28.3	23.4	20.5	
College graduate or above	27.2	19.4	12.9	
Marital Status (%)				
Married	63.9	60.9	55.0	0.010
Widowed	4.2	3.1	5.1	
Divorced	7.3	7.9	10.4	
Separated	2.4	2.3	3.2	
Never married	14.5	16.8	19.3	
Living with partner	7.7	9.1	7.0	
