# Supplementary material for: The Association between the Dietary Inflammatory Index and Thyroid Function in U.S. Adult Males
Source: Nutrients. 2021 Sep 23;13(10):3330. doi: 10.3390/nu13103330 (PMC8540204; doi:10.3390/nu13103330)
Supplement: Supplementary file 1 [file nutrients-13-03330-s001.zip › Table S2ú║The methodology of dietary inflammatory index calculation.pdf]

Supplementary material Table S2

The methodology of dietary inflammatory index calculation

Dietary inflammatory index (DII) was a literature-derived and population-based tool to assess dietary inflammatory potential via 24-h dietary recall. DII reflected both a robust literature base and standardization of individual intakes to global referent values, where 45 food parameters from 11 food consumption data sets were identified and assessed their impacts on six inflammatory markers. A total of 1943 qualified articles were screened and scored based on these 45 food parameters. If food parameters were identified to increase pro-inflammatory cytokines (TNF-α, IL-1β, IL-6, and CRP) or decrease anti-inflammatory cytokines (IL-4 and IL-10), the study was scored "+1", vice versa. Null scores were set to "0", and these values were weighted according to study design. A Z-score was generated by subtracting the "standard mean" from the amount reported and dividing this value by its standard deviation (SD). To minimize the effect of "right skewing", Z-score was converted to a percentile score. A symmetrical distribution with values centered on 0 (null) and bounded between -1(most anti-inflammatory) and +1 (most pro-inflammatory) was achieved by doubling each percentile score and then subtracting "1". This centered percentile value for each food parameter was then multiplied by its respective "overall inflammatory effect score" to obtain the "food parameter-specific DII score". Lastly, all "food parameter-specific DII scores" were added together to create the 'overall DII score' for an individual. The "overall inflammatory effect score" of 28 food parameters incorporated in our study were presented in the table below.

Food parameter	Overall inflammatory effect score
Energy (kcal)	0.18
Alcohol (g)	-0.278
Cholesterol (mg)	0.11
Caffeine (g)	-0.11
Fat (g)	0.298
Fiber (g)	-0.663
Folic Acid (μg)	-0.19
β-carotene (μg)	-0.584
Iron (mg)	0.032
Magnesium (mg)	-0.484
Zinc (mg)	-0.313
Selenium (μg)	-0.191
Thiamin (mg)	-0.098
Vitamin A (RE)	-0.401
Vitamin B 6 (mg)	-0.365
Vitamin B 12 (μg)	0.106
Vitamin C (mg)	-0.424
Vitamin D (μg)	-0.466
Vitamin E (mg)	-0.419
Protein (g)	0.021

Niacin (mg)	-0.246
Riboflavin (mg)	-0.098
Carbohydrate (g)	0.097
MUFA (g)	-0.009
PUFA (g)	-0.337
Saturated fat (g)	0.373
(n-3) Fatty acids	-0.436
(n-6) Fatty acids	-0.159
