# Supplementary figures and images for: The Association between the Dietary Inflammatory Index and Thyroid Function in U.S. Adult Males
Source: Nutrients. 2021 Sep 23;13(10):3330. doi: 10.3390/nu13103330 (PMC8540204; doi:10.3390/nu13103330)

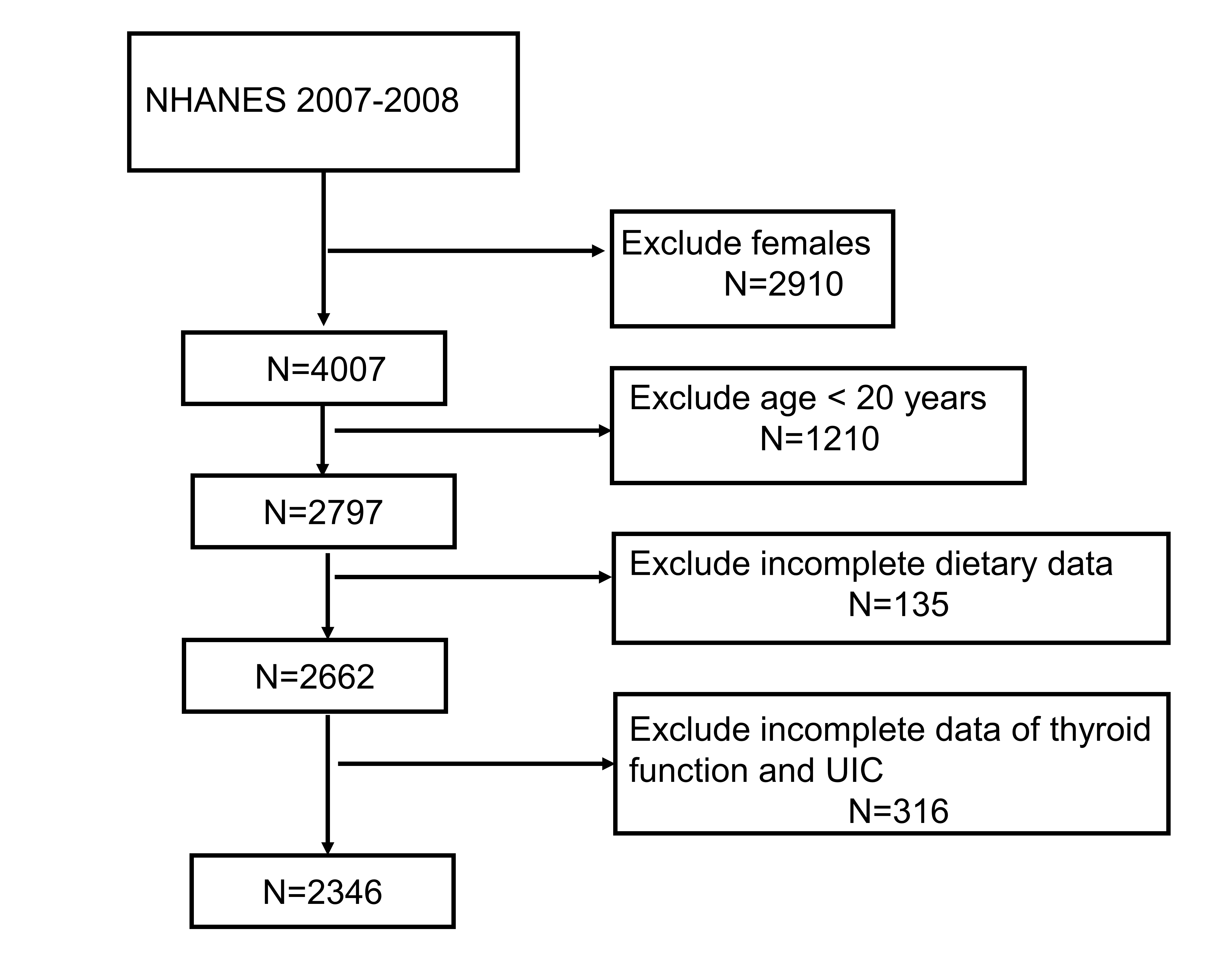

Supplement: Supplementary file 1 [file nutrients-13-03330-s001.zip › Figure S1ú║Flow diagram of the sample selection from NHANES 2007-2008.tif]
